# Orally Administered Plasmalogens Alleviate Negative Mood States and Enhance Mental Concentration: A Randomized, Double-Blind, Placebo-Controlled Trial

**DOI:** 10.3389/fcell.2022.894734

**Published:** 2022-06-02

**Authors:** Minoru Fujino, Jun Fukuda, Hirohisa Isogai, Tetsuro Ogaki, Shiro Mawatari, Atsushi Takaki, Chikako Wakana, Takehiko Fujino

**Affiliations:** ^1^ BOOCS Clinic Fukuoka, Fukuoka, Japan; ^2^ Faculty of Human Sciences, Kyushu Sangyo University, Fukuoka, Japan; ^3^ Institute of Rheological Functions of Food, Fukuoka, Japan; ^4^ Department of Integrative Physiology, Kyushu University Graduate School of Medical Sciences, Fukuoka, Japan; ^5^ The Japanese Plasmalogen Society, Fukuoka, Japan

**Keywords:** plasmalogen, scallop-derived, psychobehavioral effect, sleep, mental concentration, athlete, randomized placebo-controlled trial

## Abstract

**Background:** Plasmalogens have been shown to improve neurodegenerative pathology and cognitive function. We hypothesized that plasmalogens work in small amounts as a kind of hormone interacting with a G protein-coupled receptor, and then explored the effects of scallop-derived purified plasmalogens on psychobehavioral conditions in a randomized placebo-controlled trial of college athletes in Japan.

**Methods and materials:** Eligible participants were male students aged 18–22 years who belonged to university athletic clubs. They were randomly allocated to either plasmalogen (2 mg per day) or placebo treatment of 4 weeks’ duration. The primary outcome was the T-score of the Profile of Mood States (POMS) 2–Adult Short, and the secondary outcomes included the seven individual scales of the POMS 2, other psychobehavioral measures, physical performance, and laboratory measurements. The trial was registered at the Japan Registry of Clinical Trials (jRCTs071190028).

**Results:** Forty participants (20 in the plasmalogen group and 20 in the placebo group) completed the 4-week treatment. The Total Mood Disturbance (TMD) score of the plasmalogen group showed a greater decrease at 4 weeks than that of the placebo group while the between-group difference was marginally significant (*p* = 0.07). The anger-hostility and fatigue-inertia scores of the POMS 2 decreased significantly in the plasmalogen group, but not in the placebo group, at 4 weeks. Between-group differences in those scores were highly significant (*p* = 0.003 for anger-hostility and *p* = 0.005 for fatigue-inertia). The plasmalogen group showed a slight decrease in the Athens Insomnia Scale at 2 weeks, and the between-group difference was near-significant (*p* = 0.07). The elapsed time in minute patterns on the Uchida-Kraepelin test, which is a marker of mental concentration, revealed significantly greater performance in the plasmalogen group than in the placebo group. There were no between-group differences in physical and laboratory measurements.

**Conclusion:** It is suggested that orally administered plasmalogens alleviate negative mood states and sleep problems, and also enhance mental concentration.

## 1 Introduction

Plasmalogens are a class of glycerophospholipids containing a vinyl ether bond at the sn-1 position of the glycerol backbone and polyunsaturated fatty acids (PUFA) at the sn-2 position, being produced by the multistep process in peroxisome and endoplasmic reticulum. In humans, plasmalogens exist mainly in the forms of phosphatidyl ethanolamine plasmalogen (PlsPE) and phosphatidyl choline plasmalogen (PlsPC), of which the former is predominant in human tissues, especially in the brain (Farooqui and Horrocks, 2001; Braverman and Moser, 2012). The levels of plasmalogens have been reported to be decreased in the postmortem brain of Alzheimer’s disease (AD) and in the blood of AD patients (Ginsberg et al., 1995; Guan et al., 1999; Han et al., 2001; Goodenowe et al., 2007; Wood et al., 2010; Oma et al., 2012; Wood et al., 2015; Yamashita et al., 2016; Zarrouk et al., 2018). Several recent reviews have suggested that plasmalogen supplementation may improve cognitive function and neurodegenerative pathology (Farooqui and Horrocks, 2001; Braverman and Moser, 2012; Lizard et al., 2012; Dorninger et al., 2017; Su et al., 2019). Moreover, a simple method was developed to extract a large number of plasmalogens from animals (Mawatari et al., 2007; Mawatari et al., 2009), which enabled research on plasmalogen treatment. In mice, plasmalogen administration not only suppressed inflammation-induced activation of microglia, amyloid formation, and neuronal cell death in the brain but also improved cognitive function (Ifuku et al., 2012; Katafuchi et al., 2012; Hossain et al., 2013; Hossain et al., 2018; Gu et al., 2022; Hossain et al., 2022). A randomized controlled trial demonstrated that scallop-derived purified plasmalogens improved cognitive function in patients with mild AD or mild cognitive impairment (Fujino et al., 2017; Fujino et al., 2018). Improvement in cognitive function associated with plasmalogen supplementation was also observed in patients with moderate to severe AD (Fujino et al., 2019) and Parkinson’s disease (Mawatari et al., 2020).

Plasmalogens may also be potentially beneficial with respect to psychobehavioral aspects other than cognition. However, the effects of plasmalogen supplementation on psychological states have not been investigated while psychological distress is very common in free-living populations and affects mental and physical health (Brandt et al., 2017). Training and participation in competitive sport may affect athletes’ mood and sleep, which in turn may influence their daily life and competition performance (Weerakkody et al., 2021). We explored the effects of scallop-derived purified plasmalogens on psychobehavioral conditions of college athletes in a randomized placebo-controlled trial. The Profile of Mood States (POMS) Version 2 (Heuchert and McNair, 2012), other psychobehavioral parameters, physical performance, and laboratory measurements were used in the present study.

## 2 Methods and Materials

### 2.1 Participants

Eligible participants were unmarried male students aged 18–22 years who belonged to the athletic clubs of Kyushu Sangyo University. Those were excluded if they had regular medical treatment or follow-up in hospitals or clinics; if they participated in any other clinical trials; or if they had an allergy to shellfish. Furthermore, students were not admitted to the trial if a principal physician investigator (MF) or an organizing professor of the University (TO) deemed them ineligible to participate.

### 2.2 Design and Procedures

Study participants were recruited at the University campus *via* posters, brochures, and internet advertisements from October to November 2019. All participants gave written informed consent, and their guardians also gave consent in the case of students under 20 years of age. Eligible students were randomly allocated to either plasmalogen (2 mg per day) or placebo treatment of 4 weeks’ duration and ingested two capsules of 0.5 mg plasmalogen or placebo twice daily. Allocation was made at the baseline examination according to the sequence of a randomization list. A computer-generated list of randomizations was prepared by an independent statistician (MedStat Corporation, Fukuoka) using the permuted block method with a block size of four containing equal assignments to the two groups. The plasmalogen and placebo capsules were prepared by a manufacturer (B&S Corporation, Tokyo). Packages of plasmalogen or placebo capsules were numbered according to the list of randomizations and were transferred to the study site. The central management center confirmed the eligibility of each participant and monitored the enrollment. The list of random allocation was kept confidential until the completion of the electric data files used for the statistical analysis.

Participants visited the study site at the university campus at 0, 2, and 4 weeks for assessment of efficacy parameters and adverse events. Laboratory measurements were performed at 0 and 4 weeks of the treatment. The baseline measurements were taken on November 9–19, 2019, and the 4-week measurements were on December 9–19, 2019. Compliance with the treatment was assessed by counting the number of remaining capsules.

The primary efficacy outcome was the Total Mood Disturbance (TMD) T-score of POMS 2–Adult Short (Heuchert and McNair, 2012). Secondary outcomes included the seven individual scales of POMS 2, other psychobehavioral measures (Athens Insomnia Scale and Uchida-Kraepelin test), physical performance test (shuttle run, grip muscle strength, and standing long jump), plasmalogen levels in plasma and erythrocytes, plasma levels of brain-derived neurotrophic factor (BDNF), urinary 8-hydroxy-2′-deoxyguanosine (8-OHdG), body mass index, and percent body fat. Adverse events were captured by self-report and in-person interview if needed and by means of laboratory measurements at 4 weeks (serum biochemistry and blood cell counts).

### 2.3 Measurements

#### 2.3.1 Psychological Assessment

The POMS 2–Adult Short is a mood inventory consisting of 35 items rated on a 5-point scale from 0 (not-at-all) to 4 (extremely) to capture the seven scales measuring anger-hostility, confusion-bewilderment, depression-dejection, fatigue-inertia, tension-anxiety, vigor-activity, and friendliness during the past 1 week. The TMD was obtained by summation of the six-scale scores other than the friendliness score (Heuchert and McNair, 2012). The Japanese version of POMS 2 is commercially available (Yokoyama, 2015). We used the T-scores of the TMD and seven scales, which are standardized so that the distribution has a mean of 50 points and SD of 10 points based on a survey of Japanese adults (Yokoyama, 2015).

The Athens Insomnia Scale is a self-administered questionnaire asking eight questions rated on a 4-point scale from 0 (no problem) to 3 (severe problem) in response (Soldatos et al., 2000; Okajima et al., 2013). While the prototype is designed to assess insomnia over the past 1 month, the present study used the past 1 week as a reference period.

The Uchida-Kraepelin test is a psychodiagnostic test derived from the Kraepelin’s work curve (Kashiwagi, 1962; Seiwa, 1971), and has been used to measure work performance, and to assess occupational aptitude and mental arithmetic stressors (Sugimoto et al., 2009; Yoto et al., 2018). The standard test sheets were purchased (Japan Psychiatric Technology Institute, Inc., Tokyo). A test sheet contains an array of 17 lines of 116 random single-digit numbers per line. The task is a consecutive summation of two adjacent digits per line in one minute, the answers being written down below the line. Examinees are requested to perform calculations as quickly and accurately as possible and to move sequentially to the next line on the examiner’s cue. While the test is usually performed in two 15 min sessions with a 5 min rest, the task in the present study was one 10 min session using the first 10 lines. The number of attained calculations and incorrect answers were counted per line. Total and line-specific percentages of correct calculations were used as indices of task performance.

#### 2.3.2 Anthropometric Measurements

Height (in 0.1 cm) was measured in an upright position, and body weight (in 0.1 kg) was measured with underwear on. Body mass index (kg/m^2^) was calculated. Percent body fat was measured by the impedance method using a commercial apparatus (In Body 770, In Body Japan Co., Tokyo).

#### 2.3.3 Physical Performance Test

Physical performance was measured with respect to grip strength, standing long jump, and 20 m shuttle run test in accordance with the Manual for the New Physical Fitness Tests (age of 20–64 years) of the Japanese Ministry of Education, Culture, Sports, Science and Technology [“Ministry of Education Culture Sports Science and Technology. Manual for the New Physical Fitness Tests (Age of 20-64 Years),” 1999]. The number of repeats in the shuttle run test was transformed to VO_2_ max by using the conversion table of the Manual.

#### 2.3.4 Laboratory Measurements

Venous blood was drawn after an overnight fast, and urine sample was collected before blood sampling. Plasmalogen levels in plasma and erythrocyte membrane were determined at the Institute of Rheological Functions of Food (Hisayama-machi, Fukuoka) according to the method described elsewhere (Mawatari et al., 2007; Mawatari et al., 2016). Plasma BDNF and urinary 8-OHdG and deoxyguanosine (dG) were determined at the Department of Integrative Physiology, Kyushu University Graduate School of Medical Sciences. Plasma BDNF was determined by the ELISA method (Tiekou Lorinczova et al., 2020) using a commercial kit (biosensis, Adelaide). Urinary 8-OHdG and dG were measured using a commercial kit by the TAS system (TAS Project Co., Ltd., Fukuoka) (Matsuoka et al., 2010). 8-OHdG and dG were detected by electrochemical and UV detector at 254 nm followed by HPLC, using the same sample. Hereby, 8-OHdG/dG ratio indicating biological oxidation was calculated. Routine biochemical measurements and blood cell counting were carried out at an external laboratory (BML, Tokyo). Adverse events in laboratory measurements were defined in accordance with the Common Terminology Criteria Adverse Events (CTCAE) version 5.0 (“U.S. Department of Health and Human Service. Common Terminology Criteria for Adverse Events (Ctcae) Version 5.0 Published: November 27, 2017”).

### 2.4 Materials

The lipid composition of purified ether phospholipids from scallop is shown in Table 1. One capsule, used in this study, contained 0.48 mg of ethanolamine plasmalogen and 0.02 mg of choline plasmalogen.

**TABLE 1:** Lipid composition of purified ether phospholipid from scallop.

Lipid	(mg/g)
Ethanolamine plasmalogen	39.7
Ethanolamine alkyl phospholipid	2.9
Choline plasmalogen	1.6
Choline alkyl phospholipid	32.6
Cholesterol	11.5
Ceramide aminoethyl phosphonate	10.6

### 2.5 Statistical Analysis

The sample size was determined to be 20 in each group. On the basis of the results from randomized and non-randomized trials of psychobehavioral therapy or supplementation on POMS (Katsuki et al., 2013; Okuyama et al., 2018; Irie et al., 2019), we assumed a 20% greater improvement in the POMS T-score for the plasmalogen group than for the placebo group. The SD of the TMD T-score is 10, and the SD of the change in the T-score is 10 if the correlation between the T-scores before and after treatment is assumed to be 0.50 (Follmann et al., 1992). Under the conditions of a two-sided significance level of 0.05 and detection power of 0.80, the required sample size was calculated as 17 for each group. The target number was set to be 20 for each as a precaution against dropouts.

The safety analysis population consisted of all randomized participants who received at least one dose of treatment and completed both baseline and follow-up assessments regarding adverse events. The efficacy analysis population comprised those in the safety analysis population for whom efficacy data were available.

Mean and standard deviation (SD) were presented for continuous variables, and proportion was used for dichotomous variables. The changes from baseline at 2 and 4 weeks were compared between the two groups. The magnitude of the between-group difference in the change from baseline was expressed by mean difference and 95% confidence interval (CI). The between-group difference was assessed by unpaired *t*-test for continuous variables and by Fisher’s exact probability for dichotomous variables. Statistical significance of the change from baseline in each group was assessed by paired *t*-test. The interaction between baseline value and treatment was evaluated by using multiple regression analysis. The analysis on the interaction had not been specified in the protocol; however, it was adopted because it was naturally conceivable that improvement, if any, could be expected in men having a poor state of the outcome at baseline. The effects on daily physical and mental conditions were examined by the mixed-model regression analysis, in which days of assessment were nested in individuals. Statistical significance was declared if the two-sided *p* was <0.05. Statistical analyses were carried out by Stata Statistical Software Release 13 (StataCorp, College Station, TX).

## 3 Results

A total of 42 participants were randomly allocated to either plasmalogen (*n* = 21) or placebo (*n* = 21) treatment, all of which completed the 4-week treatment. All the participants consumed more than 80% of the capsules containing the test substance in each group. However, the efficacy analysis included 40 participants (20 in the plasmalogen group and 20 in the placebo group) because the Review Board recommended that the analysis should exclude the two last-enrolled students who exceeded the target number specified in the protocol.

### 3.1 Baseline Characteristics

There was no measurable between-group difference with respect to background characteristics of the study participants such as age, smoking, alcohol drinking, and anthropometric measurements (Table 2). None of the primary and secondary outcome variables showed an appreciable difference between the two groups at baseline (Table 3). Mean T-scores for the scales of vigor-activity and friendliness were approximately 60 points while mean T-scores were not more than 50 points for the TMD and the other five individual scales.

**TABLE 2:** Background characteristics of the study participants.

Variable	Plasmalogen (*n* = 20)	Placebo (*n* = 20)	*p* ^*^
Age (year), mean ± SD	19.6 ± 0.7	19.3 ± 0.9	0.23
Current smoking, n (%)	0 (0.0)	2 (10.0)	0.49
Alcohol use (≥1/week), n (%)	0 (0.0)	3 (15.0)	0.23
Food allergy, n (%)	2 (10.0)	2 (10.0)	1.00
Height (cm), means ± SD	170.3 ± 5.2	171.5 ± 5.0	0.47
Body weight (kg), means ± SD	66.8 ± 9.2	68.1 ± 5.6	0.59

SD: standard deviation.

*Unpaired t-test for continuous variables and Fisher’s exact test for dichotomous variables.

**TABLE 3:** Primary and secondary outcomes at baseline lightfaced (means ± SD).

Variable	Plasmalogen (*n* = 20)	Placebo (*n* = 20)	*p* ^a^
POMS2 T-score^b^			
Total mood disturbance	46.4 ± 7.6	44.3 ± 7.5	0.39
Anger-hostility	46.8 ± 8.8	43.5 ± 6.3	0.17
Confusion-bewilderment	49.3 ± 8.8	48.4 ± 7.0	0.72
Depression-dejection	47.6 ± 6.5	46.1 ± 6.8	0.49
Fatigue-inertia	48.4 ± 7.1	46.4 ± 6.5	0.36
Tension-anxiety	47.1 ± 8.6	46.4 ± 8.9	0.79
Vigor-activity	57.2 ± 7.7	58.0 ± 7.7	0.73
Friendliness	59.7 ± 10.5	59.6 ± 11.2	0.97
Other psychological tests			
Athens Insomnia Scale^b^	3.3 ± 2.5	3.1 ± 2.4	0.73
Uchida-Kraepelin Test	47.6 ± 11.8	43.0 ± 13.3	0.26
Physical fitness test			
Grip strength (kg)	43.7 ± 8.5	44.9 ± 6.4	0.60
Standing long jump (cm)	234 ± 19	224 ± 29	0.22
VO_2_max (ml/kg/min)	49.0 ± 4.0	49.0 ± 3.2	0.99
Obesity-related measurement			
Body mass index (kg/m^2^)	23.0 ± 2.7	23.2 ± 2.0	0.81
Percent body fat (%)	14.8 ± 5.1	14.3 ± 6.0	0.79
Laboratory measurement			
Plasma PlsPE (mg/dl)	5.15 ± 1.32	5.27 ± 2.06	0.83
Erythrocyte PlsPE (%)	8.54 ± 0.87	8.54 ± 0.75	0.99
Plasma BDNF (ng/ml)	8.52 ± 3.74	7.81 ± 2.41	0.48
Urinary 8-OHdG (ng/ml)	39.4 ± 24.1	58.7 ± 85.7	0.34
Urinary 8-OHdG/dG (%)	2.98 ± 2.46	2.91 ± 3.20	0.94

BDNF, brain-derived neurotrophic factor; dG, deoxyguanosine; 8-OHdG, 8-hydoroxy deoxyguanosine; PlsPE, phosphatidyl ethanolamine plasmalogen; SD, standard deviation.

a Unpaired t-test.

b *n* = 19 in the plasmalogen group.

### 3.2 Effects on POMS 2

**FIGURE 1:**
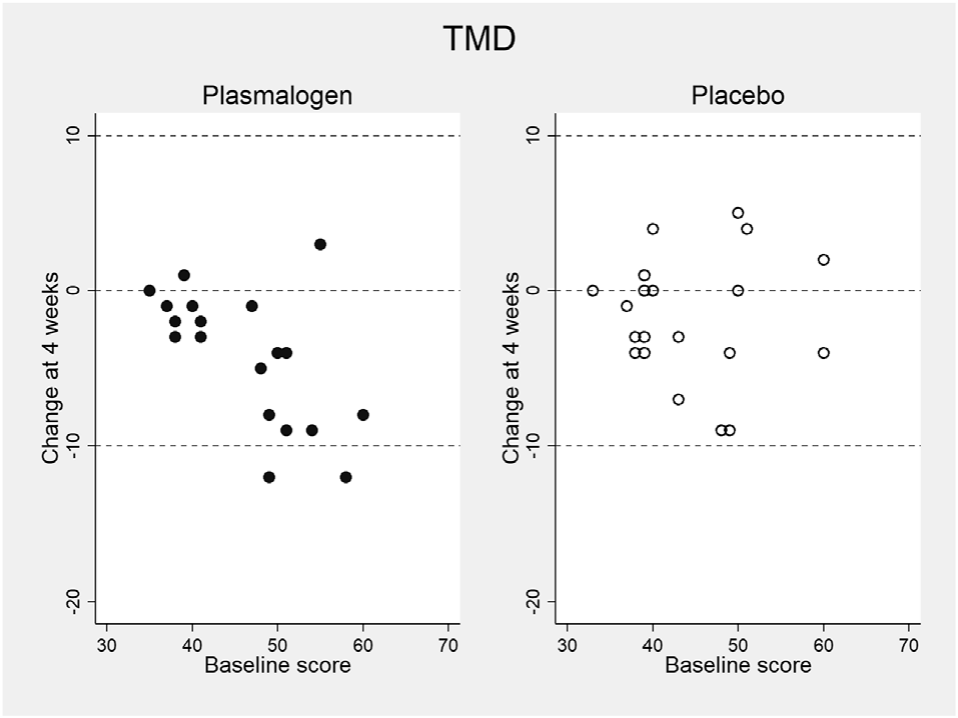
Change in total mood disturbance (TMD) T-score of POMS 2 at 4 weeks versus baseline score in plasmalogen and placebo groups.

Table 4 shows the changes from baseline in T-scores of the TMD and the seven scales of POMS 2 at 2 and 4 weeks. Notable changes were not observed for these scores at 2 weeks. The TMD T-score at 4 weeks decreased statistically significantly in the plasmalogen group (*p* < 10^−3^) and decreased near-significantly in the placebo group (*p* = 0.07), resulting in a greater decrease in the former group than in the latter. While the overall between-group difference in the decrease was not statistically significant (*p* = 0.07), the decrease in the plasmalogen group was more evident in those with higher baseline scores (interaction *p* = 0.048), as illustrated in Figure 1.

**TABLE 4:** Changes of POMS 2 T-scores from baseline at 2 and 4 weeks in plasmalogen and placebo groups.

Scale	Plasmalogen		Placebo		Difference		
	Mean ± SD	*p* ^a^	Mean ± SD	*p* ^a^	Mean	95% CI	*p* ^b^
2 weeks	(*n* = 18)		(*n* = 19)				
Total mood disturbance	−1.5 ± 3.8	0.12	−0.6 ± 5.2	0.60	−0.9	−3.9, 2.2	0.57
Anger-hostility	−2.4 ± 5.0	0.06	0.5 ± 6.1	0.71	−2.9	−6.7, 0.8	0.12
Confusion-bewilderment	−2.8 ± 6.0	0.07	−1.0 ± 7.7	0.58	−1.8	−6.4, 2.8	0.44
Depression-dejection	−0.1 ± 4.8	0.92	0.3 ± 5.3	0.80	−0.4	−3.8, 3.0	0.80
Fatigue-inertia	−0.6 ± 5.7	0.65	1.1 ± 6.8	0.49	−1.7	−5.9, 2.5	0.41
Tension-anxiety	−2.6 ± 4.6	0.03	−1.8 ± 5.5	0.17	−0.8	−4.2, 2.6	0.63
Vigor-activity	−1.4 ± 7.1	0.42	2.6 ± 7.7	0.16	−4.0	−9.0, 0.9	0.11
Friendliness	−3.5 ± 10.5	0.17	1.4 ± 7.0	0.39	−0.9	−10.8, 1.0	0.10
4 weeks	(*n* = 19)		(*n* = 20)				
Total mood disturbance	−4.2 ± 4.3	<10^−3^	−1.8 ± 4.0	0.07	−2.5	−5.2, 0.2	0.07
Anger-hostility	−4.0 ± 4.3	<10^−3^	0.3 ± 4.1	0.75	−4.3	−7.0, −1.6	0.003
Confusion-bewilderment	−4.9 ± 6.9	0.006	−2.0 ± 5.5	0.13	−3.0	−7.0, 1.0	0.14
Depression-dejection	−1.8 ± 5.9	0.19	−0.2 ± 3.1	0.77	−1.6	−4.7, 1.4	0.28
Fatigue-inertia	−6.4 ± 6.1	<10^−3^	−0.9 ± 5.4	0.49	−5.5	−9.2, −1.8	0.005
Tension-anxiety	−6.0 ± 5.6	<10^−3^	−3.4 ± 6.1	0.02	−2.6	−6.4, 1.2	0.17
Vigor-activity	−3.7 ± 8.8	0.09	2.5 ± 9.4	0.26	−6.1	−12.1, −0.2	0.04
Friendliness	−6.4 ± 14.2	0.07	0.4 ± 7.5	0.81	−6.8	−14.1, 0.5	0.07

CI: confidence interval; SD: standard deviation.

a Paired *t*-test for the within-group comparison.

b Unpaired *t*-test for the between-group comparison.

The scores of anger-hostility and fatigue-inertia decreased significantly in the plasmalogen group, but not in the placebo group, at 4 weeks. The decreases were statistically significantly greater in the plasmalogen group than in the placebo group (*p* = 0.003 for anger-hostility and *p* = 0.005 for fatigue-inertia). The vigor-activity score also showed a statistically significant difference in the 4-week change between the two groups (*p* = 0.04) whereas the within-group change was not statistically significant in either group; the T-score tended to decrease in the plasmalogen group and to increase in the placebo group. There was no measurable difference in the changes in the other scales between the two groups.

**FIGURE 2:**
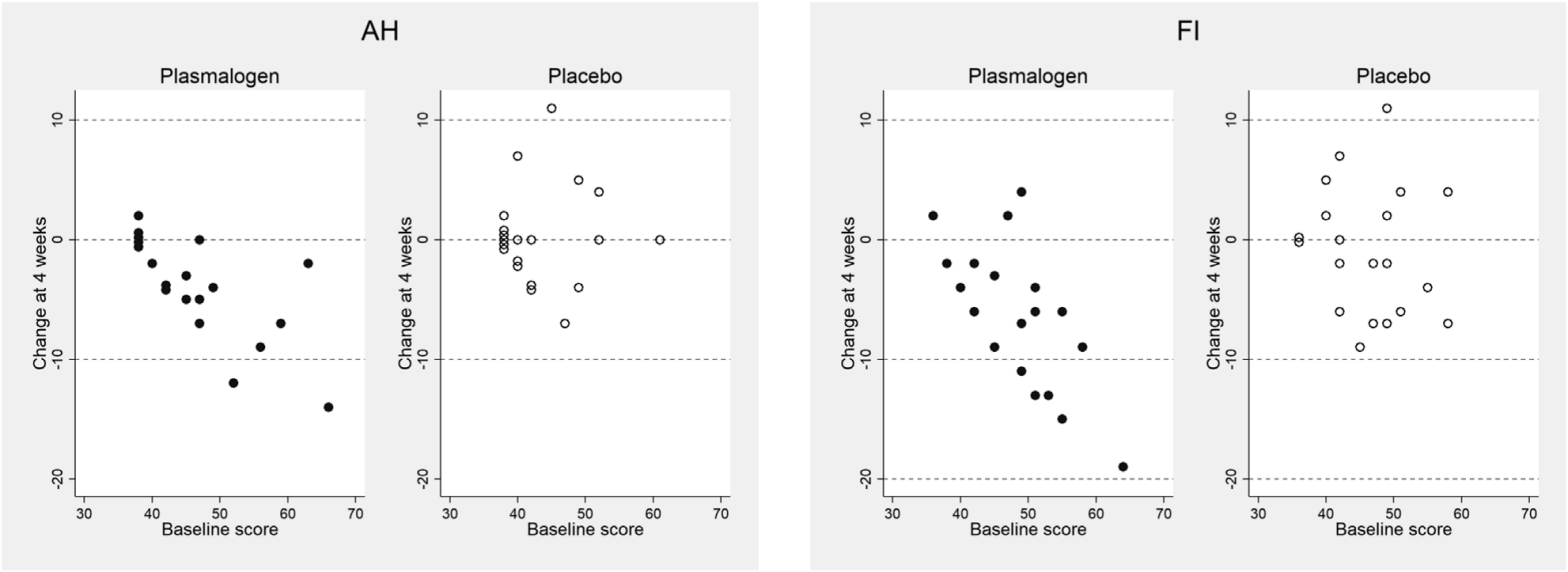
Changes in anger-hostility (AH) and fatigue-inertia (FI) T-scores of POMS 2 at 4 weeks versus baseline scores in plasmalogen and placebo groups. Points of identical values in the two dimensions are minimally shifted along the vertical direction and are displayed as aggregated circles.

*P* values for the interactions between baseline score and treatment on the changes in the T-scores at 4 weeks were as follows: anger-hostility 0.03, confusion-bewilderment 0.12, depression-dejection 0.24, fatigue-inertia 0.07, tension-anxiety 0.19, vigor-activity 0.11, and friendliness 0.74. The decreases in the scores of anger-hostility and fatigue-inertia in the plasmalogen group were greater when the baseline scores were higher (Figure 2).

### 3.3 Effects on Other Psychological Parameters

Table 5 summarizes the results of the Athens Insomnia Scale and Uchida-Kraepelin test. The Plasmalogen group showed small, but statistically significant decreases in the Athens Insomnia Scale score at both 2 and 4 weeks whereas no such decreases were noted in the placebo group; the between-group difference in the decrease was nearly significant at 2 weeks (*p* = 0.07), but not at 4 weeks (*p* = 0.26). However, the decrease in the plasmalogen group was more pronounced in those with higher baseline scores at 2 weeks (interaction *p* = 0.02) and 4 weeks (interaction *p* = 0.004).

**TABLE 5:** Changes from baseline in Athens Insomnia Score and Uchida-Kraepelin Test (achievement percentage) at 2 and 4 weeks.

Scale	Plasmalogen		Placebo		Difference		
	Mean ± SD (*n*)	*p* ^a^	Mean ± SD (*n*)	*p* ^a^	Mean	95% CI	*p* ^b^
2 weeks							
Athens Insomnia Score	−1.4 ± 2.1 (19)	0.009	−0.2 ± 1.9 (20)	0.64	−1.2	−2.5, 0.1	0.07
Uchida-Kraepelin Test	9.5 ± 4.9 (20)	<10^−7^	7.2 ± 4.9 (20)	<10^−5^	2.3	−0.9, 5.5	0.15
4 weeks							
Athens Insomnia Score	−1.3 ± 2.4 (19)	0.03	−0.5 ± 1.7 (20)	0.21	−0.8	−2.1, 0.6	0.26
Uchida-Kraepelin Test	11.8 ± 6.7 (20)	<10^−6^	8.9 ± 5.4 (20)	<10^−6^	2.9	−1.0, 6.8	0.14

CI: confidence interval; SD: standard deviation.

a Paired *t*-test for the within-group comparison.

b Unpaired *t*-test for the between-group comparison.

**FIGURE 3:**
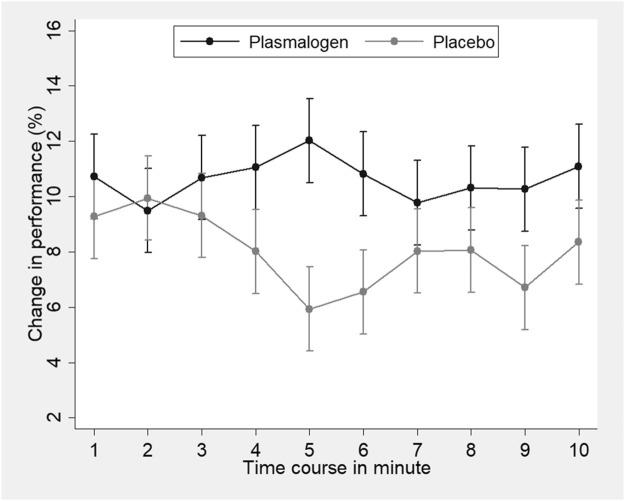
Change in the Uchida-Kraepelin test performance according to time-course minutes of the task in plasmalogen and placebo groups. Based on repeated measures analysis of variance with weeks of treatment (2 points) and elapsed minutes (10 points) specified as repeated measures. Vertical bars represent 95% confidence intervals of marginal means. Interaction for treatment and elapsed minutes was statistically significant (*p* < 0.01, corrected by the Greenhouse-Geisser method).

A highly significant improvement in the Uchida-Kraepelin test was observed at 2 and 4 weeks in both groups. The improvement seemed to be slightly greater in the plasmalogen group than in the placebo group, but the between-group difference was not significant at 2 and 4 weeks, respectively. The baseline values did not affect the treatment effect either at 2 weeks (interaction *p* = 0.85) or at 4 weeks (interaction *p* = 0.65). The post-hoc analysis showed a significant interaction between treatment and elapsed time in minute on the Uchida-Kraepelin test (*p* < 0.01). The Plasmalogen group showed a significantly increased performance in the middle (5 and 6 min) and toward the end of the 10 min task compared with the placebo group (Figure 3).

### 3.4 Physical Performance and Obesity-Related Measures

There was no measurable effect of plasmalogen supplementation on either physical performance or obesity-related measures (Table 6).

**TABLE 6:** Changes in physical fitness, laboratory measurements, and obesity-related measures at 2 and 4 weeks from baseline.

	Plasmalogen (n = 20)		Placebo (n = 20)		Difference		
	Mean ± SD	*p* ^a^	Mean ± SD	*p* ^a^	Mean	95% CI	*p* ^b^
Physical fitness test							
Grip strength (kg)	−0.8 ± 3.1	0.27	−1.4 ± 2.1	0.01	0.6	−1.1, 2.3	0.51
Standing long jump (cm)	−3.4 ± 10.2	0.16	5.7 ± 19.6	0.21	−9.1	−19.3, 1.1	0.08
VO_2_max (ml/kg/min)	0.5 ± 2.6	0.40	0.0 ± 2.2	0.95	0.5	−1.1, 2.1	0.53
Laboratory measurement							
Plasma PlsPE (mg/dl)	−0.84 ± 0.89	<10^−3^	−0.62 ± 1.66	0.11	−0.22	−1.07, 0.64	0.61
Erythrocyte PlsPE (%)	0.64 ± 0.57	<10^−4^	0.76 ± 0.57	<10^−5^	−0.12	−0.48, 0.25	0.52
Plasma BDNF (ng/ml)	0.01 ± 4.32	0.99	0.14 ± 3.80	0.87	−0.13	−2.73, 2.48	0.92
Urinary 8-OHdG (ng/ml)^c^	−0.171 ± 0.656	0.26	0.005 ± 1.054	0.98	−0.176	−0.738, 0.386	0.53
Urinary 8-OHdG/dG^c^	−0.430 ± 0.875	0.04	−0.080 ± 0.900	0.69	−0.350	−0.918, 0.219	0.22
Obesity-related measure							
Body mass index (kg/m^2^)	0.22 ± 0.41	0.03	0.18 ± 0.35	0.04	0.05	−0.20, 0.29	0.71
Percent body fat (%)	0.93 ± 1.42	0.008	0.83 ± 1.57	0.03	0.10	−0.86, 1.06	0.83

CI: confidence interval; SD: standard deviation.

a Paired *t*-test for the within-group comparison.

b Unpaired *t*-test for the between-group comparison.

c Measurements were transformed to the natural logarithm scale.

### 3.5 Laboratory Outcomes

Plasma PlsPE levels significantly decreased in the plasmalogen group (*p* < 10^−3^) and non-significantly in the placebo group (*p* = 0.11), and the decrease did not differ by treatment group (Table 6). On the other hand, erythrocyte PlsPE increased to almost the same magnitude in the plasmalogen (*p* < 10^−4^) and the placebo (*p* < 10^−5^) groups, with no between-group difference in the change of PlsPE. Plasma BNDF, urinary 8-OHdG, and urinary 8-OHdG/dG did not change differentially in the plasmalogen and placebo groups. Nor did body mass index and percent body fat.

### 3.6 Adverse Events

Clinical adverse events were reported by three participants (common cold, acute gastroenteritis, and calcaneal fracture) in the plasmalogen group and by one participant (common cold) in the placebo group (*p* = 0.61). Laboratory adverse events were noted in 14 men (66.7%) in the plasmalogen group and in 13 men (61.9%) in the placebo group (*p* = 1.00). The average numbers of the laboratory adverse events were 1.1 (SD 1.0) in the plasmalogen group and 1.3 (SD 1.5) in the placebo group (*p* = 0.63). Details of laboratory adverse events are described in Supplementary Table S1. Almost all of the laboratory adverse events were grade 1. The most common one was an elevation in serum creatine phosphokinase (CPK).

## 4 Discussion

In male college athletes, a 4-week supplementation with plasmalogens improved the T-scores of anger-hostility and fatigue-inertia more greatly than with placebo and also tended to alleviate the overall mood disturbance. The findings suggest that plasmalogens may mitigate a mood state of anger-hostility and enhance the perception of recovery from fatigue-inertia. It is of particular interest that the effects of plasmalogens on the TMD and the scales of anger-hostility and fatigue-inertia were greater in those with worse scores at baseline. These interactions further support the beneficial effect of plasmalogens on mood state.

The finding on the vigor-activity score was an unexpected one. The vigor-activity score decreased by 3.7 points in the plasmalogen group and increased by 2.5 points in the placebo group. While these changes were not statistically significant in either group, the plasmalogen group statistically significantly deteriorated the vigor-activity scale in comparison with the placebo group. The vigor-activity scale represents a positive mood state and seems to be the opposite mood of fatigue-inertia. The vigor-activity scale represents the degree of being “lively,” “active,” “energetic,” “vigorous” and “enthusiastic,” whereas the fatigue-inertia scale measures the degree of being “worn out,” “fatigued,” “exhausted,” “weary” and “drained” (Heuchert and McNair, 2012). In a previous study of government employees in the United States (Travis et al., 2018), a psychological intervention of meditation resulted in an increase in the POMS vigor-activity score and consistent decreases in the five other negative scales. In that study, the Pearson correlation coefficient for the 4-month changes in the scores between the vigor-activity and fatigue-inertia scales was −0.52 (Travis et al., 2018). In the present study, the vigor-activity score was weakly correlated with the fatigue-inertia score negatively at baseline and in a positive direction longitudinally (*r* = −0.13 for the baseline score and *r* = 0.17 for the change at 4 weeks). The decrease in the vigor-activity score may not necessarily indicate an unfavorable effect on the present study population. It deserves to be mentioned that the vigor-activity scores were within normal limits at both baseline and 4 weeks. A small decrease of four points in the vigor-activity score may have reflected a change toward mental calmness and composedness or weakening of aggressiveness and offensiveness in the young athletes with a relatively high score at baseline.

The findings regarding the Athens Insomnia Scale suggest that plasmalogens may alleviate sleep problems. It is noteworthy that the effect of plasmalogens decreasing the score of the Athens Insomnia Scale was greater in those with higher baseline scores, consistently at 2 and 4 weeks. It is also notable that the effect of plasmalogens was observable even at 2 weeks of treatment.

A learning or practice effect has been documented in the Uchida-Kraepelin test, particularly when the test is repeated at short intervals (Seiwa, 1971). It is thus likely that the successive increases in the performance at 2 and 4 weeks are ascribed to a learning effect. It is known that the performance decreases with the passage of time in a session of the Uchida-Kraepelin test (Kashiwagi, 1962). It is thus of interest to examine the treatment effect on minute-specific performance. Increased performance in the plasmalogen group was more notable in the middle (5 and 6 min) and toward the end of the 10-min task (Figure 3). To our knowledge, none of the previous intervention studies have addressed the effect on the minute-specific performance in the Uchida-Kraepelin test (Ataka et al., 2008; Johnson et al., 2016; Takanari et al., 2016; Saito et al., 2018). It could be argued that plasmalogens may maintain the performance in the middle of work, elicit the so-called last spurt in work performance, and increase mental concentration. The present findings are compatible with the abovementioned effect of plasmalogens improving the POMS fatigue-inertia score.

It seems strange that a small dose (2 mg per day) of plasmalogens showed the prominent improvement and enhancement in brain function as mentioned above although the same small dose of plasmalogens also revealed the significant efficacy on cognitive function in AD, mild cognitive impairment, and Parkinson’s disease. One of the major mechanisms for such effects is that plasmalogens may be the ligand of G protein-coupled receptors and thus may act as a hormone. More detailed mechanisms have been discussed in the previous report (Fujino et al., 2017; Fujino et al., 2020).

In this study, all participants were young athletes and there was no improvement in physical performance measured by grip strength, standing long jump, and 20 m shuttle run. However, some improvement in competition performance might be expected because it is well known that competition performance is strongly affected by such negative mood states, sleep disorders, and mental concentration (Mah et al., 2011; Brandt et al., 2017; Weerakkody et al., 2021) as improved in this study. Further research is required to determine a positive correlation between plasmalogens and competition performance.

Despite the abovementioned significant improvement in psychobehavioral measures in the plasmalogen group, plasmalogen blood levels (plasma PlsPE and erythrocyte PlsPE) showed no significant difference between the two groups. The reason for that is not clear. However, it might be partly due to the fact that the study period was as short as 1 month, considering that there was no significant between-group difference in plasmalogen blood levels during the first 2 months after administration shown in the previous study (Fujino et al., 2017).

Recent studies have demonstrated that oxidative stress-induced neuroinflammation impairs brain function including cognitive function. However, the present study did not find a significant change in urinary 8-OHdG, known to reflect oxidative stress (Valavanidis et al., 2009; Urbaniak et al., 2020). Moreover, no statistically significant change was observed in blood BDNF although plasmalogens are assumed to improve brain function by increasing BDNF (Hossain et al., 2022). These results suggest that neither urinary 8-OHdG nor blood BDNF may reflect a minute change in the brain.

This study strongly suggests that oral administration of plasmalogens alleviates negative mood states and sleep disorders, and enhances mental concentration. These effects may be responsible for the suppression effects of plasmalogens on oxidative stress-induced neuroinflammation (Ifuku et al., 2012; Hossain et al., 2018). Yet, the fact also remains that most of the neuroinflammation-suppression effects observed in animal studies were achieved using mouse hippocampus, providing no direct evidence of suppression effects in the amygdala or neocortex related to the abovementioned symptoms and function. As another potential mechanism for these effects, it may be suggested that plasmalogens improved sleep conditions, resulting in the enhancement of mental concentration.

There are several weaknesses to be noted in this study although random allocation, use of placebo, and high compliance to test substances are among the strengths of the present study. The 4 week treatment period may have been relatively short, and the number of study participants was too small to detect an observed between-group difference in terms of the TMD score (the primary outcome) which turned out to be much smaller than expected. Further studies are needed to corroborate the observed effects of plasmalogens on negative mood states of the POMS 2, sleep disorders, and mental concentration in large and different populations such as insomnia and depression.

## Data Availability

The raw data supporting the conclusion of this article will be made available by the authors, without undue reservation.

## Supplementary Material

Supplementary Table S1 Laboratory adverse effects in plasmalogen and placebo groups.
