# Supplementary material for: Orally Administered Plasmalogens Alleviate Negative Mood States and Enhance Mental Concentration: A Randomized, Double-Blind, Placebo-Controlled Trial
Source: Front Cell Dev Biol. 2022 Jun 2;10:894734. doi: 10.3389/fcell.2022.894734 (PMC9203127; doi:10.3389/fcell.2022.894734)
Supplement: Supplementary file 1 [file Table1.docx]

**SUPPLEMENTARY TABLE 1** Laboratory adverse effects in plasmalogen and placebo groups

|  |  | Number |  |  |
| --- | --- | --- | --- | --- |
| Laboratory adverse effect^a^ | Grade | Plasmalogen | Placebo | *p*^b^ |
| Fasting plasma glucose, decrease | 1 | 0 | 1 | 1.00 |
| Serum total cholesterol, decrease | 1 | 4 | 2 | 0.66 |
| Serum LDL cholesterol, decrease | 1 | 2 | 1 | 1.00 |
| Serum triglycerides, decrease | 1 | 2 | 3 | 1.00 |
| Serum total protein, increase | 1 | 1 | 1 | 1.00 |
| Serum A/G ratio, increase | 1 | 1 | 3 | 0.61 |
| Serum total bilirubin, increase | 1 | 0 | 1 | 1.00 |
| Serum AST, increase | 1 | 1 | 1 | 1.00 |
| Serum amylase, increase | 1 | 1 | 2 | 1.00 |
| Serum CPK, increase^c^ | 1, 2, 4 | 4 | 5 | 1.00 |
| Serum BUN, increase | 1 | 1 | 1 | 1.00 |
| Serum uric acid, increase | 1 | 1 | 1 | 1.00 |
| Serum total cholesterol, increase | 1 | 0 | 1 | 1.00 |
| Serum LDL cholesterol, increase | 1 | 1 | 1 | 1.00 |
| Serum HDL cholesterol, increase | 1 | 2 | 1 | 1.00 |
| Serum CRP, increase | 1 | 1 | 0 | 1.00 |
| Erythrocytes, increase | 1 | 0 | 1 | 1.00 |
| Hematocrit, increase | 1 | 1 | 1 | 1.00 |
| Leukocytes, increase | 1 | 0 | 1 | 1.00 |
| Platelets, increase | 1 | 1 | 0 | 1.00 |

A/G: albumin/globulin; AST: aspartate aminotransferase; BUN: blood urea nitrogen; CPK: creatine phosphokinase; CRP: C-reactive protein; HDL: high-density lipoprotein; LDL: low-density lipoprotein.

^a^Based the change at 4 weeks compared with baseline

^b^Fisher’s exact test.

^c^CPK increase of grade 2 numbered 1 in each group, and increase of grade 4 numbered 1 in plasmalogen group.
